# Supplementary material for: Proteomic signatures of smoking and their associations with risk of incident diseases and mortality in diverse populations
Source: Nat Commun. 2025 Dec 24;17:928. doi: 10.1038/s41467-025-67656-x (PMC12830939; doi:10.1038/s41467-025-67656-x)
Supplement: Supplementary file 1 — Supplementary Information [file 41467_2025_67656_MOESM1_ESM.pdf]

Supplementary Materials for

Proteomic signatures of smoking and their associations with risk of incident diseases and mortality in diverse populations

Sihao Xiao[1,2,3]*, Bowen Liu[1], M. Austin Argentieri[4,5], Lazaros Belbasis[1], Claire L. Shovlin[6,7], Jennifer A. Collister[1], Siyi Wang[8], Eilis Hannon[8], Jun Liu[1], Kahung Chan[1], Rami Muath Mosaoa[9,3], Liming Li[10,11,12], Jun Lv[10,11,12], Canqin Yu[10,11,12], Dianjianyi Sun[10,11,12], Jonathan Mill[8], Robert Clarke[1], David J. Hunter[1,5], Derrick Bennett[1], Alejo J. Nevado-Holgado[2,3,13], Zhengming Chen[1], Najaf Amin[1], Cornelia van Duijn[1,2,3] *

*Corresponding author. Email:sihao.xiao@bnc.ox.ac.uk,cornelia.vanduijn@ndph.ox.ac.uk

The file includes:

Supplementary Figure 1 to 15

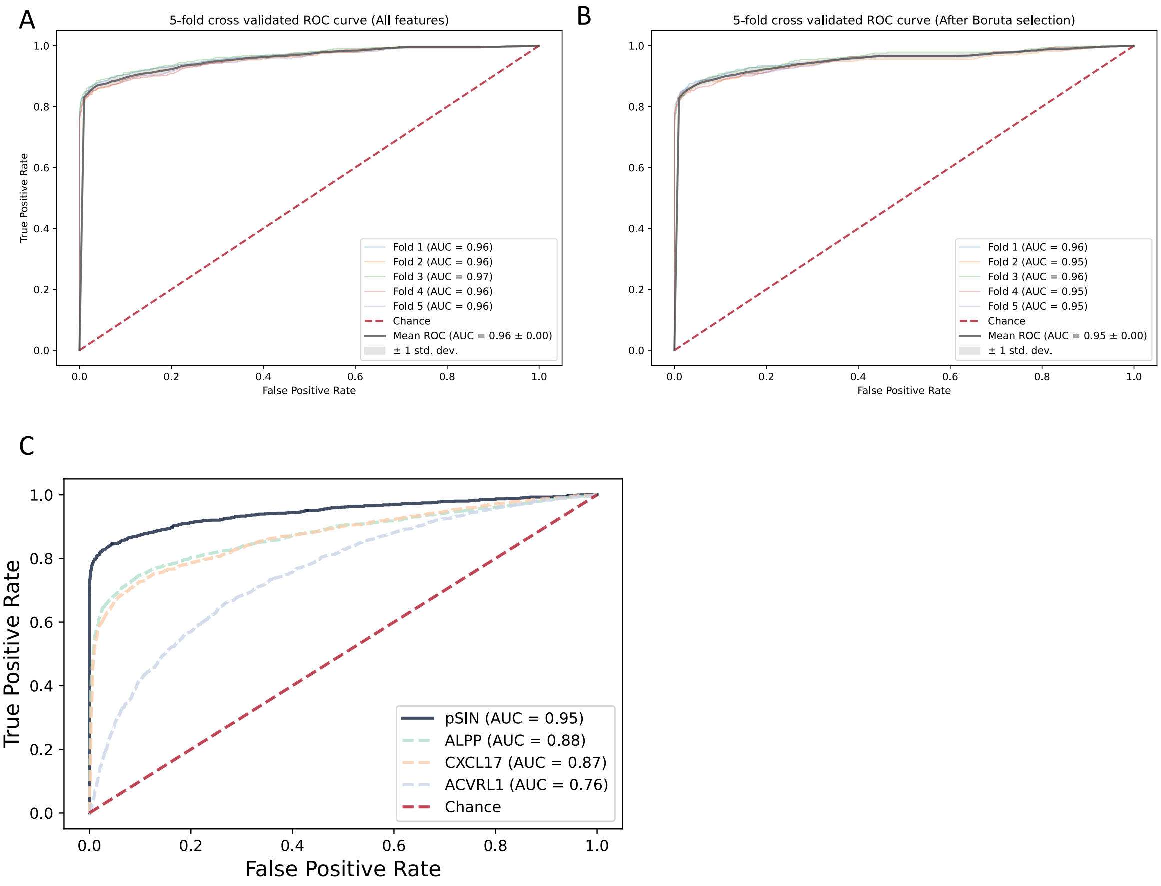

Supplementary Figure 1 Performance of the classification model.
a)5-fold cross validated ROC curve of models trained using all proteins and b) 5-fold cross validated ROC curve of models trained using Boruta selected 51 proteins only c) ROC curve comparison of the gradient boosting model comprising 51 proteins in the UKB test dataset comparing to the performance of the top3 single protein when differentiating current smokers from never smokers.

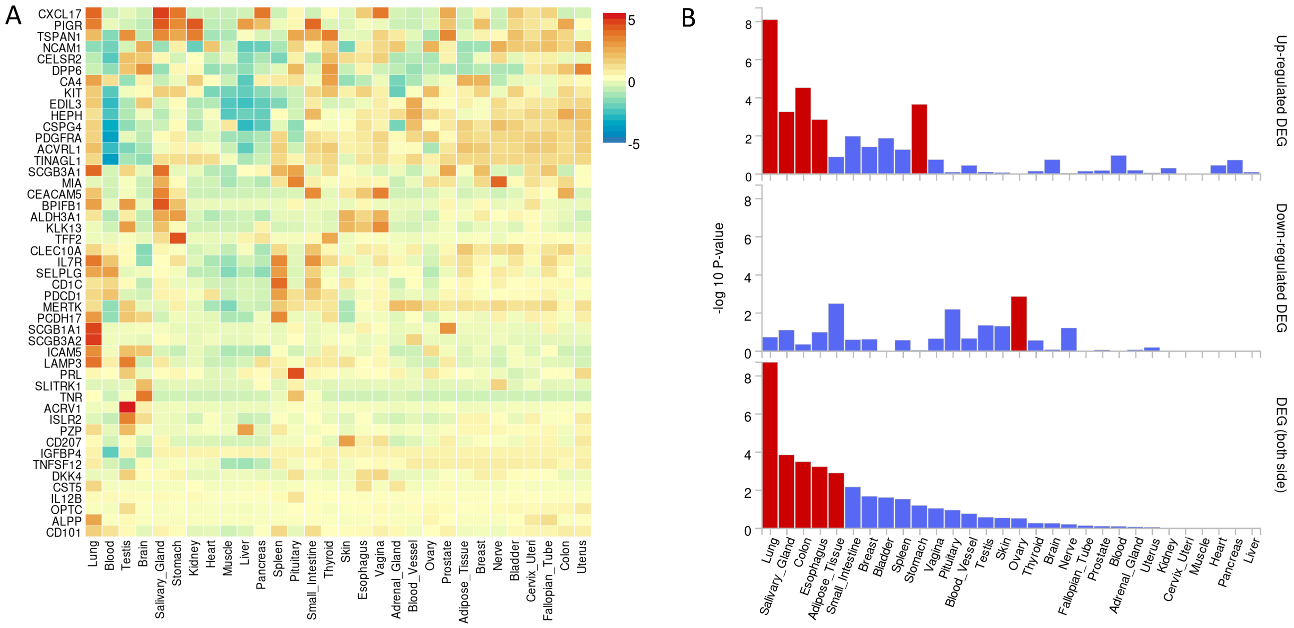

Supplementary Figure 2 *Tissue specific expression of the Boruta selected proteins.*
Tissue specific protein expression data was extracted from the Genotype-Tissue Expression (GTEx) project2 database v.8 and the heatmap was plotted using FUMAGWAS webtool. Values showed on the heatmap were average of normalized expression per gene. Differential expression genes (DEG) were identified by 2-sided t-test per tissue type versus all other tissue types. Genes with a Bonferroni corrected p-value < 0.05 and absolute log fold change > 0.58 were selected as DEG and were shown as red color. a) heatmap of average of normalized expression per gene per tissue. X-axis denotes the corresponding tissues and Y-axis denotes each of the selected proteins. b) differential expression genes (DEG) were identified by 2-sided t-test per tissue type versus
all other tissue types. Genes with a Bonferroni corrected p-value < 0.05 and absolute log fold change > 0.58 were selected as DEG and were shown as red color

A

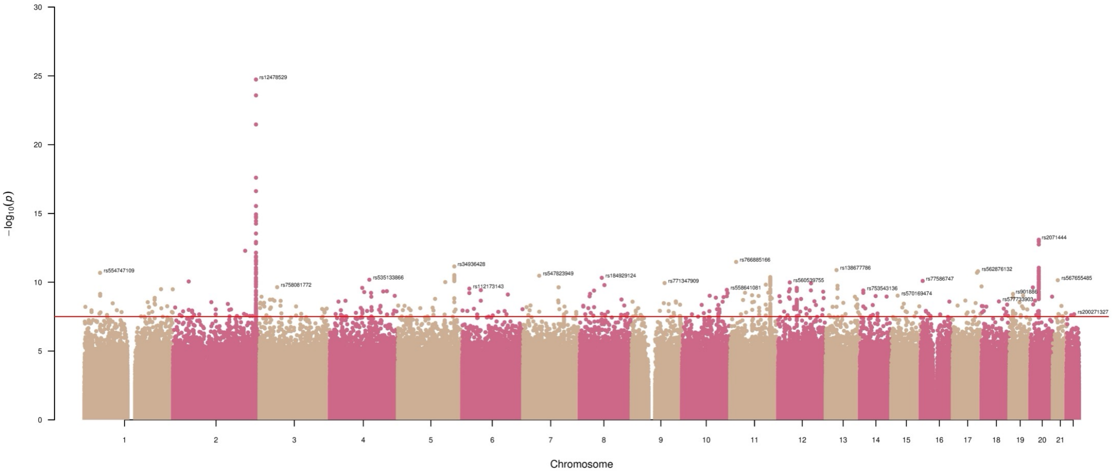

B

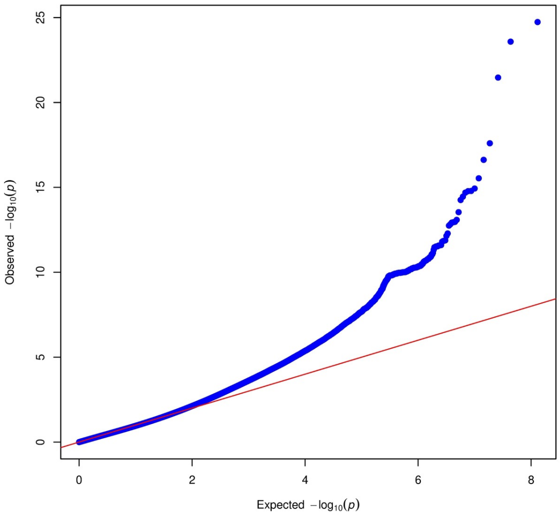

Supplementary Figure 3 Results of GWAS results on pSIN.

Results of GWAS adjusted for age, sex and 40 PCs a) Manhattan plot shows the p-value between SNPs and pSIN. A cut-off p-value of $5*10^{-8}$ was used and shown as red line in the plot. Lead significant SNPs were annotated. b) shows the Quantile-quantile plot (QQ-plot) of GWAS analysis

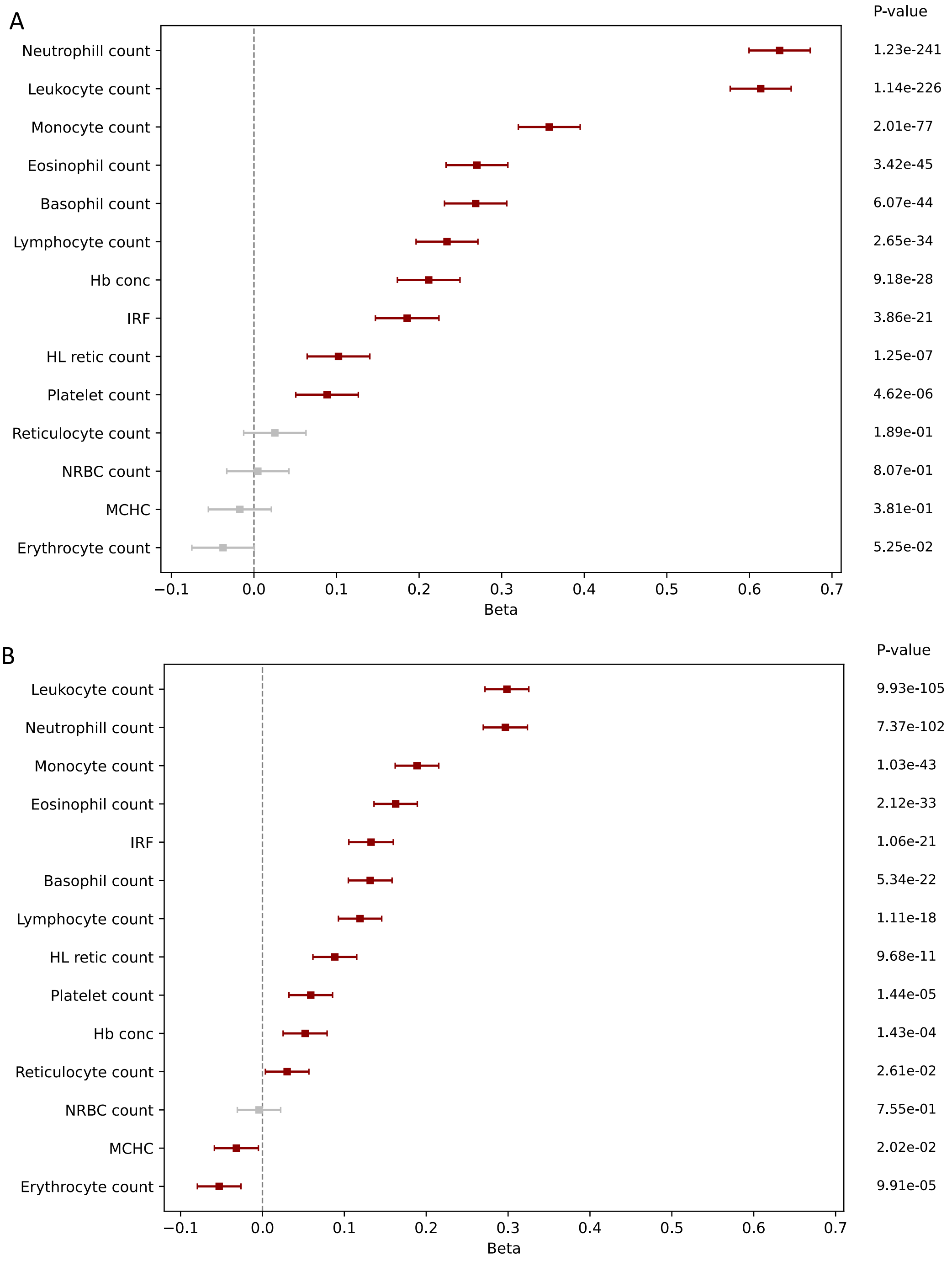

Supplementary Figure 4 haematological measurements showed significant association with pSIN.

A) Linear regression analysis between individual haematological measurements and pSIN was performed within the whole UKB population adjusting for recruitment center, Townsend deprivation index, IPAQ physical activity group, ethnicity, alcohol frequency, and education. Beta values with 95% confidence intervals were shown. Red colour denotes the association is significant after correcting for FDR multiple testing. Hb, haemoglobin, IRF, immature reticulocyte fraction; HL retic count, reticulocyte (red blood cell) count; NRBC, nucleated red blood cells; MCHC, mean corpuscular haemoglobin concentration. B) Model adjust additionally for smoking status.

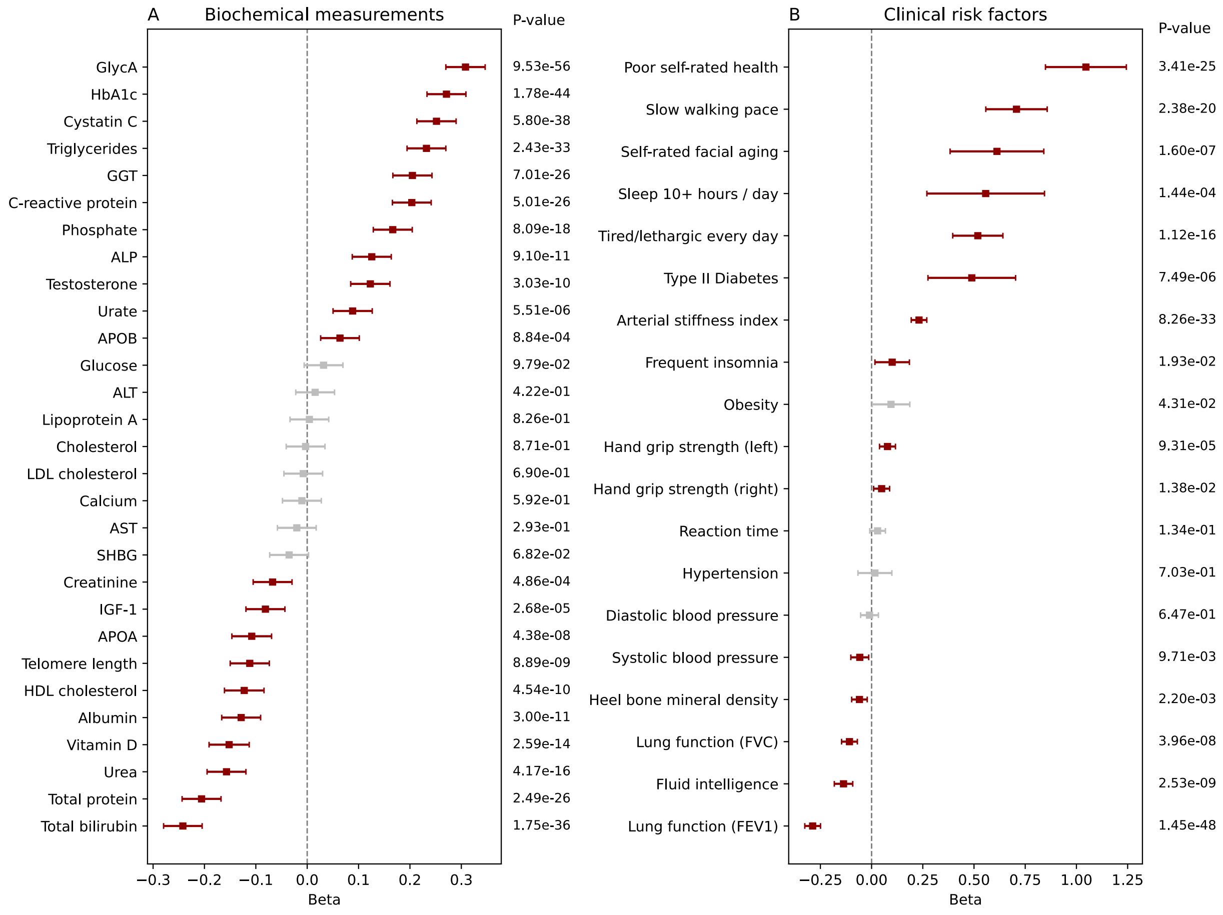

Supplementary Figure 5 Relationship between blood biomarkers and clinical risk factors with pSIN. Linear regression analysis was performed within the whole UKB population adjusting for recruitment center, Townsend deprivation index, IPAQ physical activity group, ethnicity, alcohol frequency, and education. Beta values with 95% confidence intervals were shown. Red colour denotes the association is significant after correcting for FDR multiple testing. a) Association between biochemical measurements and pSIN. b) Association between clinical risk factors and pSIN.

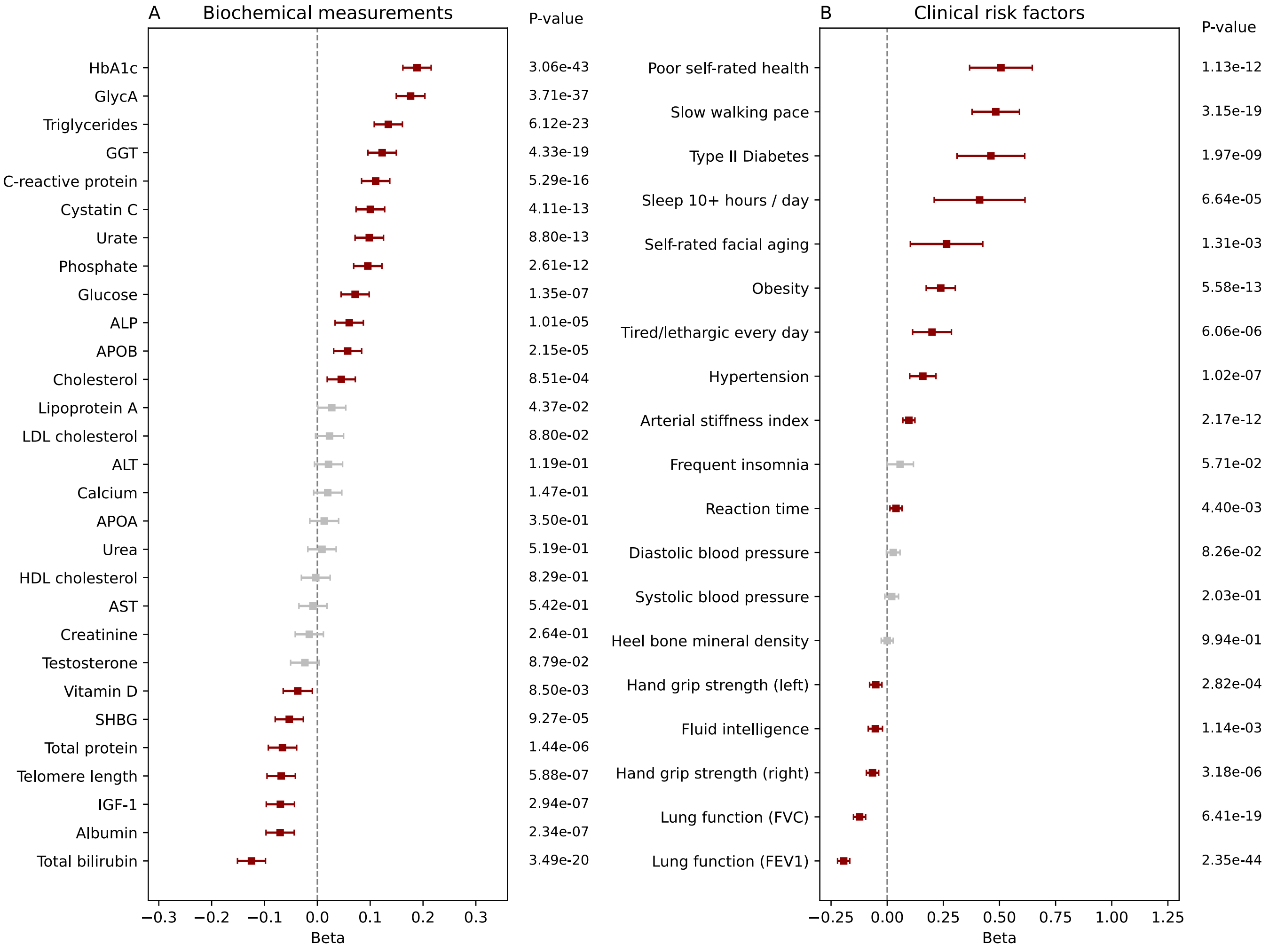

Supplementary Figure 6 Relationship between blood biomarkers and clinical risk factors with pSIN independent of smoking status.

Linear regression analysis was performed within the whole UKB population adjusting for recruitment center, Townsend deprivation index, IPAQ physical activity group, ethnicity, alcohol frequency, education and smoking status. Beta values with 95% confidence intervals were shown. Red colour denotes the association is significant after correcting for FDR multiple testing. a) Association between biochemical measurements and pSIN. b) Association between clinical risk factors and pSIN.

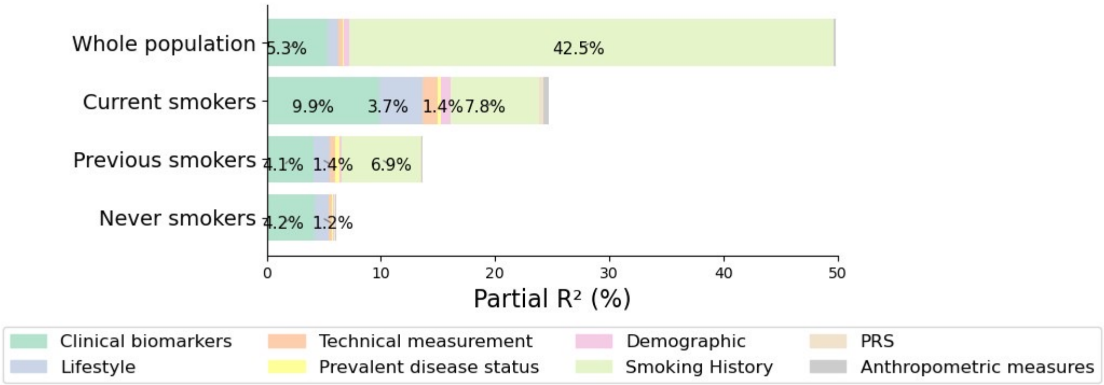

Supplementary Figure 7 Relative contribution of each category to pSIN when competing in one model.

Linear regression followed by ANOVA was performed to study the contribution of each category to pSIN stratified by smoking status. Partial R^2 was shown, and the exact percentage was annotated if it is larger than 1%. For smoking history, smoking status and pack years were included for the whole population, pack years, smoking years, tobacco type, number of cigarettes per day were included for current smokers, pack years, smoking years, smoking cessation years, tobacco type, number of cigarettes per day were included for previous smokers, and passive smoking exposure was included for never smokers.

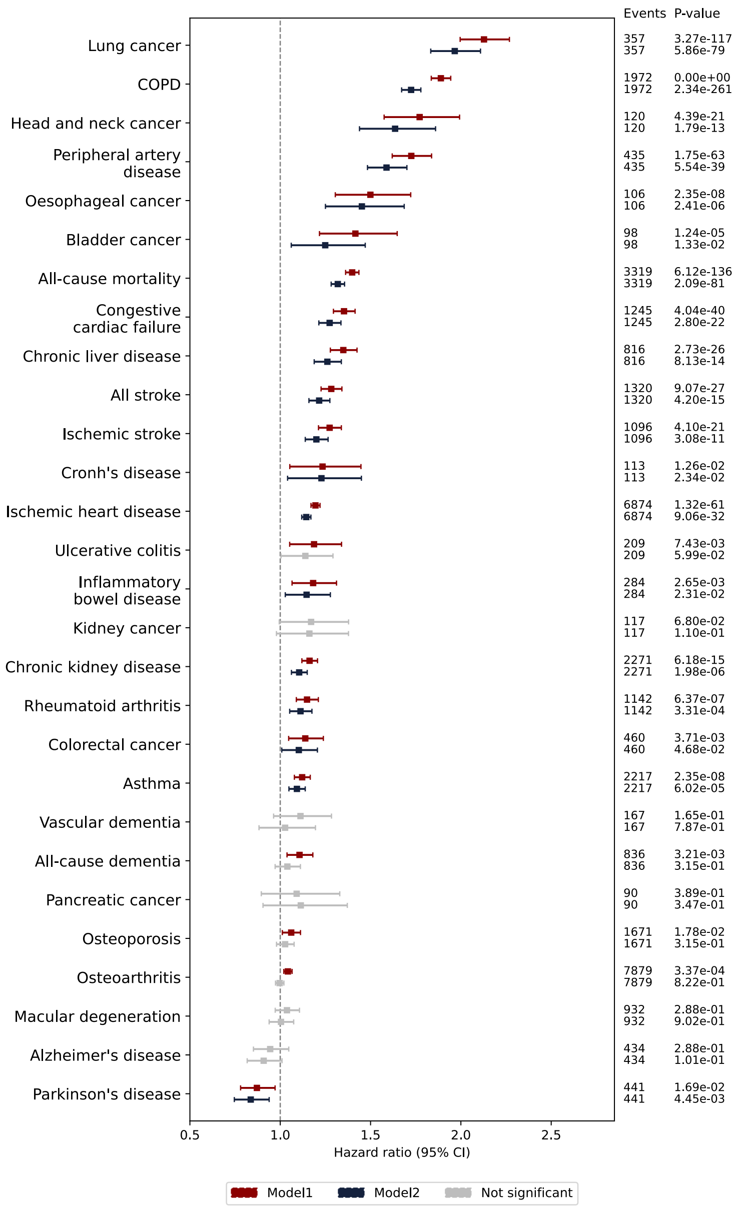

Supplementary Figure 8 pSIN is associated with future risks of morbidities and mortality. Forest plot shows association between pSIN and 24 morbidities and mortality which has at least 80 cases during follow-up time using multi-variate cox proportional hazard model. In model 1, the exposure was pSIN without adjusting for any covariate as age and sex already been regressed out in protein level. Model 2 was adjusted for recruitment centre, Townsend deprivation index, IPAQ physical activity group, ethnicity, alcohol frequency, BMI, and education years. Hazard ratios with 95% confidence intervals were shown. P-values were corrected for FDR multiple testing and non-significant associations were shown as grey colour.

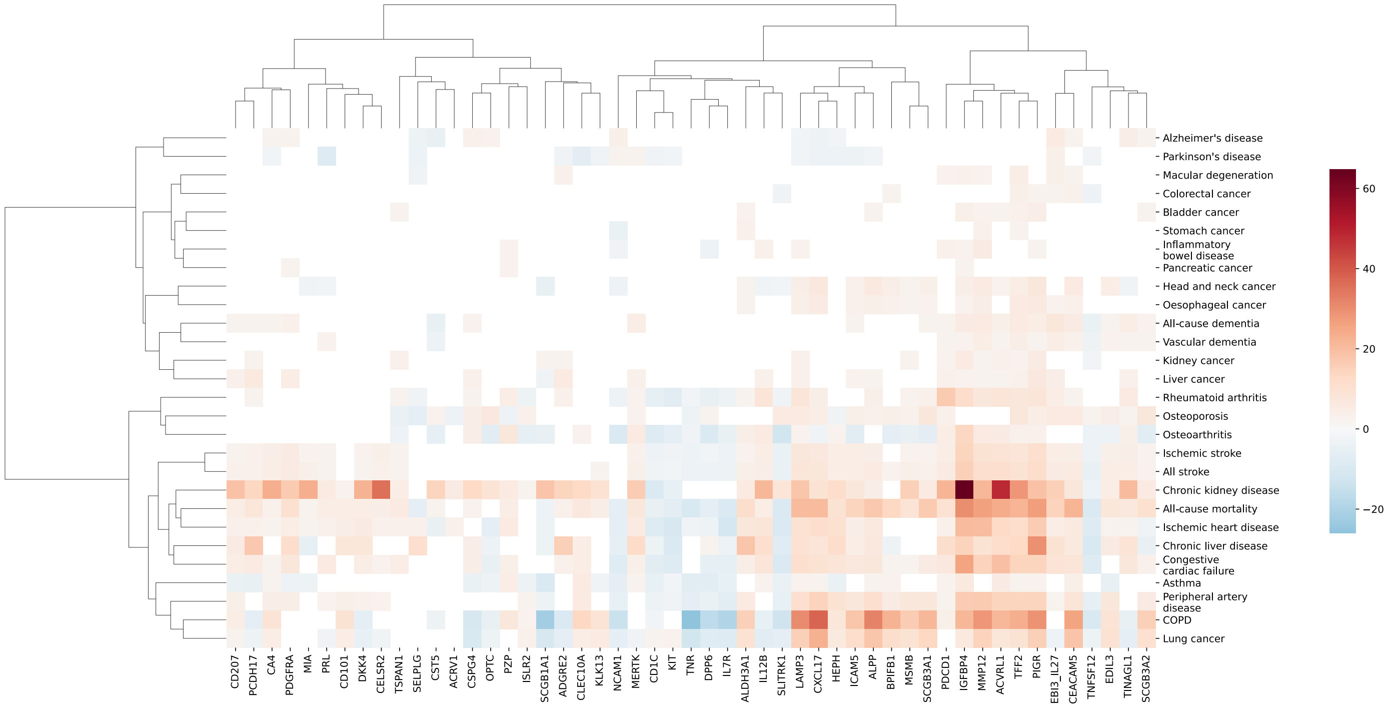

Supplementary Figure 9 Association between individual proteins and major diseases, and mortality.
COX proportional hazard model was used to assess the association between each of the 51 protein and diseases and mortality. Model was adjusted for recruitment centre, ethnicity, education years, and Townsend deprivation index. Z-score was shown on the heatmap. Association p value was corrected for FDR multiple testing and none-significant associations were shown as white colour.

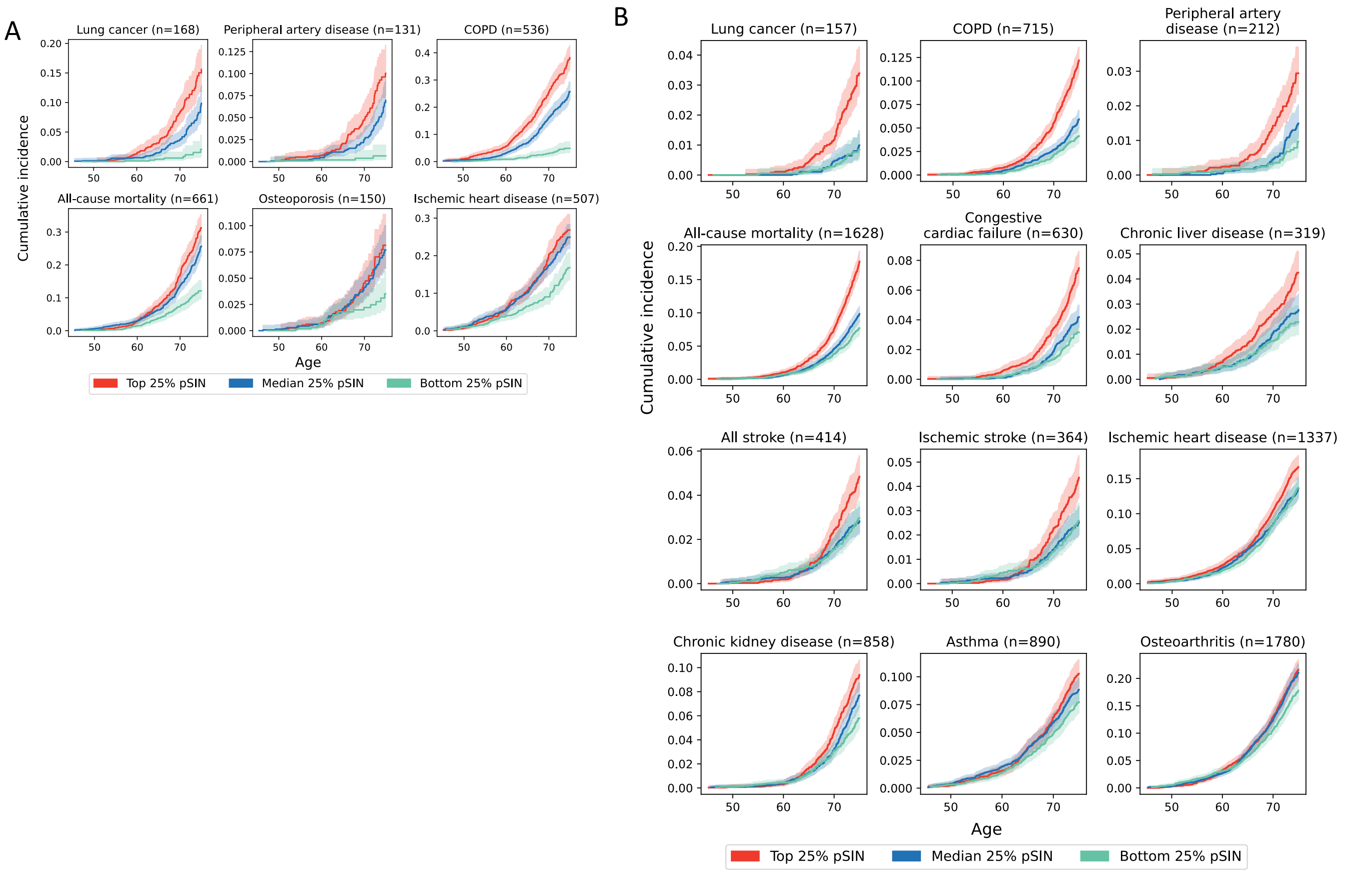

Supplementary Figure 10 pSIN differentiates future risks of morbidities and mortality in current and previous smokers.

a) shows cumulative incidence plot of top, median and bottom 25% of the pSIN in the current smokers with 95% confidence interval shown as lighter shading. X-axis denotes the chronological age and Y-axis denotes the cumulative incidence. Cumulative incidence and number at risk at each age point is shown in Supplementary Data 17 and Supplementary Data 18. b) shows cumulative incidence plot of top, median and bottom 25% of the pSIN in the previous smokers with 95% confidence interval shown as lighter shading. Cumulative incidence and number at risk at each age point is shown in Supplementary Data 20 and Supplementary Data 21. Only outcomes that were significant in all three cox models were displayed here.

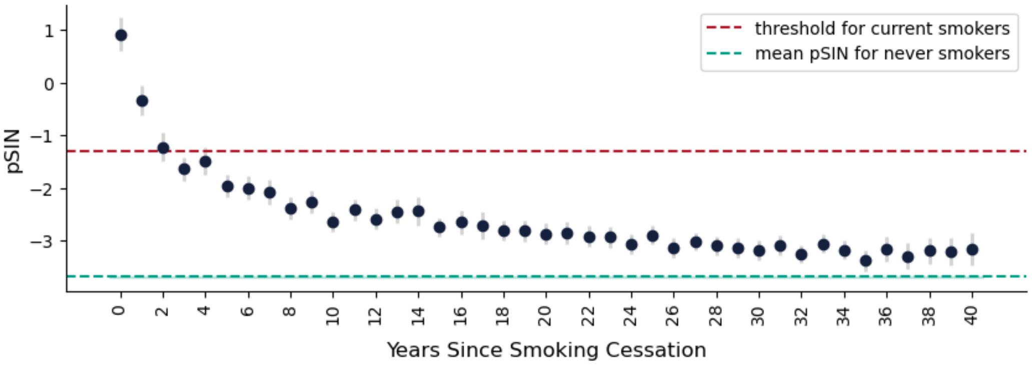

Supplementary Figure 11 Mean pSIN by years since smoking cessation. Mean pSIN in each year since cessation bin was shown in the plot with 95%CI. Red dashed line shows the threshold of differentiating current smoker from never smokers with FPR of 0.05. Green dashed line shows the mean pSIN of self-reported never smokers with 95%CI shown in green shadow.

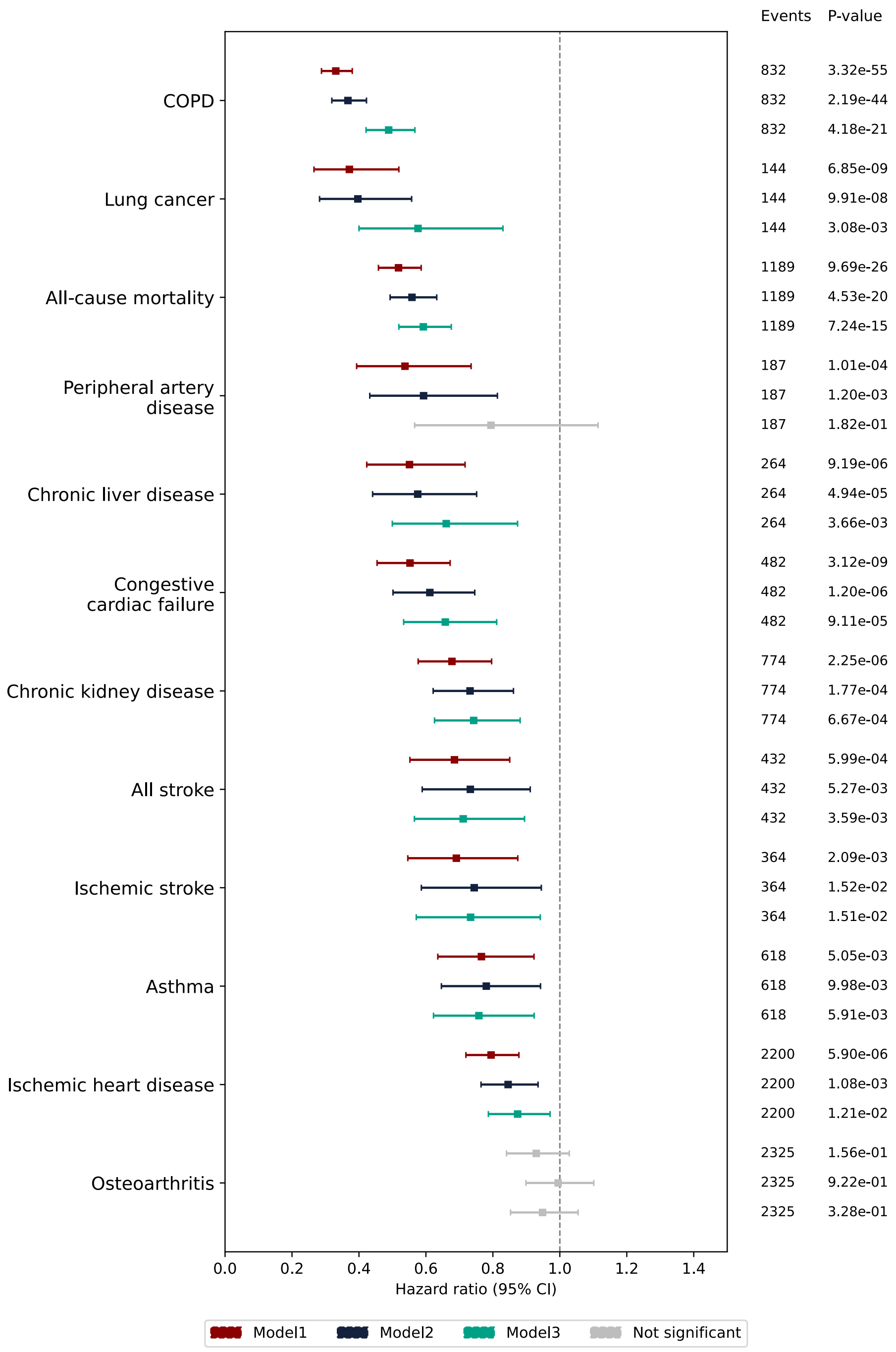

Supplementary Figure 12 previous smokers with pSIN similar to never smokers were associated with lower morbidity and mortality risks.

Forest plot shows association between low-risk group in previous smokers and health outcomes which has at least 80 cases during follow-up time and were significant in previous smoker model using multi-variate cox proportional hazard model. The comparisons are made between previous smokers with low-risk based on pSIN (i.e., those whose proteomic profiles resemble never smokers) and high-risk based pSIN. In model 1, the exposure was pSIN without adjusting for any covariate as age and sex already been regressed out in protein level. Model 2 was adjusted for recruitment centre, Townsend deprivation index, IPAQ physical activity group, ethnicity, alcohol frequency, BMI, and education years. Model 3 further adjusted for smoking pack years and smoking cessation time. Hazard ratios with 95% confidence intervals were shown. P-values were corrected for FDR multiple testing and non-significant associations after corrections were shown as grey colour.

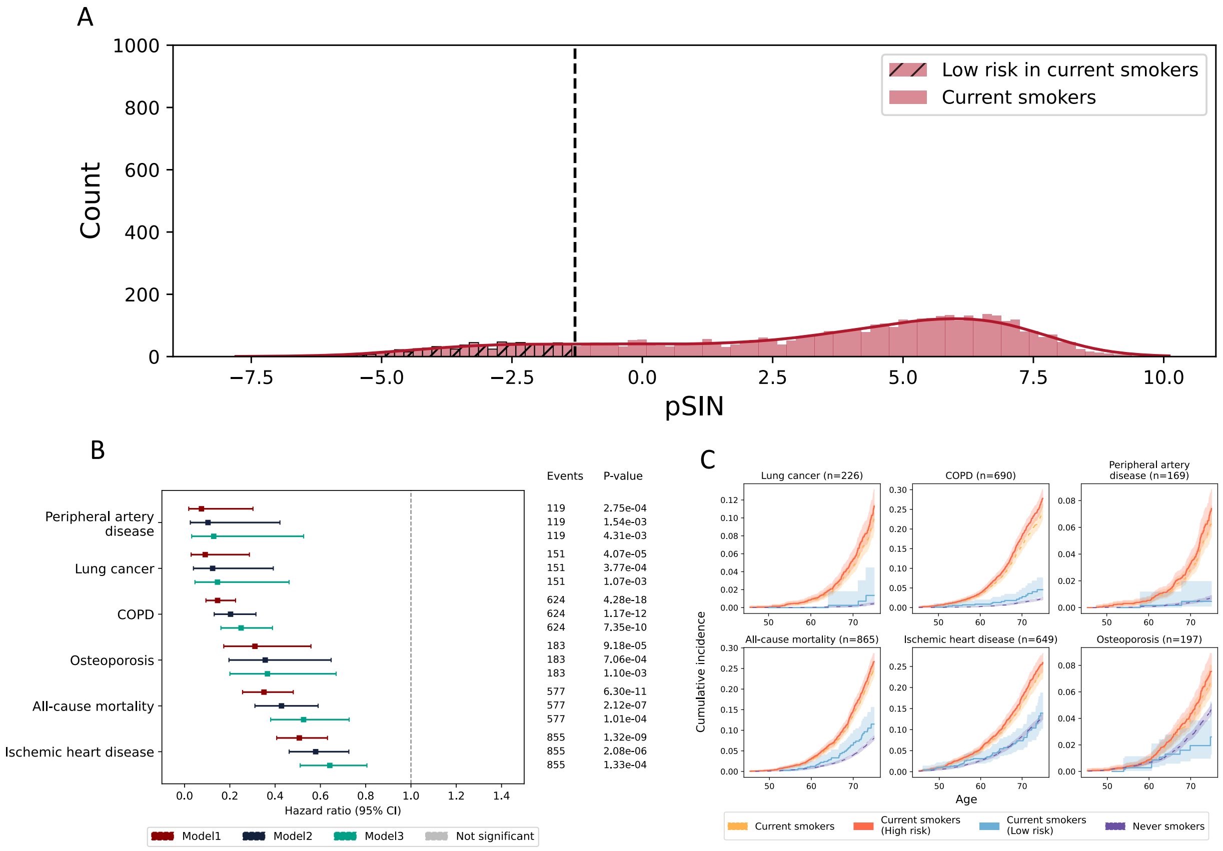

Supplementary Figure 13 people in current smokers with pSIN similar to never smokers were associated with lower morbidity and mortality risks.

(a) shows the distribution of pSIN in current smokers. Dotted line denotes the cut-off when differentiating current smokers from never smokers at FPR of 0.05 dividing current smokers into two groups. Hashed part denotes the group in current smokers with a similar pSIN as never smokers. (b) Forest plot shows association between low-risk group in current smokers and 5 morbidities and mortality which has at least 80 cases during follow-up time and were significant in current smoker model using multi-variate cox proportional hazard model. In model 1, the exposure was pSIN without adjusting for any covariate as age and sex already been regressed out in protein level. Model 2 was adjusted for recruitment centre, Townsend deprivation index, IPAQ physical activity group, ethnicity, alcohol frequency, BMI, and education years. Model 3 further adjusted for smoking pack years. Hazard ratios with 95% confidence intervals were shown. P-values were corrected for FDR multiple testing and non-significant associations after corrections were shown as grey colour. (c) shows cumulative incidence plot of low and high-risk group defined by pSIN in current smokers (orange and blue) with self-reported current smokers as positive control (yellow) and self-reported never smokers as negative control (purple). Cumulative incidence and number at risk charts are shown in Supplementary Data 27, 28 respectively.

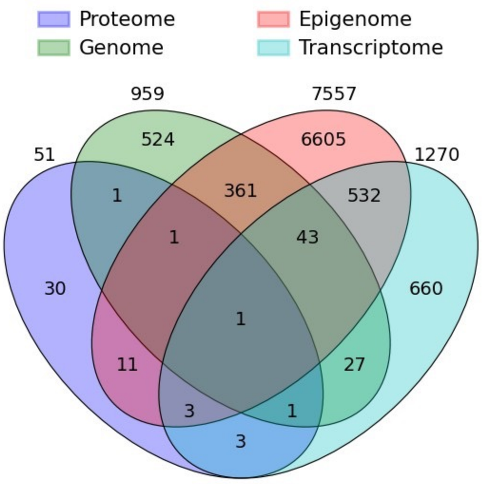

Supplementary Figure 14 Over lapping genes selected by pSIN and major genomic, transcriptomic and epigenomic studies.

pSIN selected 51 proteins were compared against three major studies each for genomic, transcriptomic and epigenomic wide association study of smoking initiation. 30 proteins were not found in previous omics study. Comparing against individual studies of biomarkers of smoking (also including protein biomarkers) 15 of the proteins selected by pSIN were not seen in the previous studies.

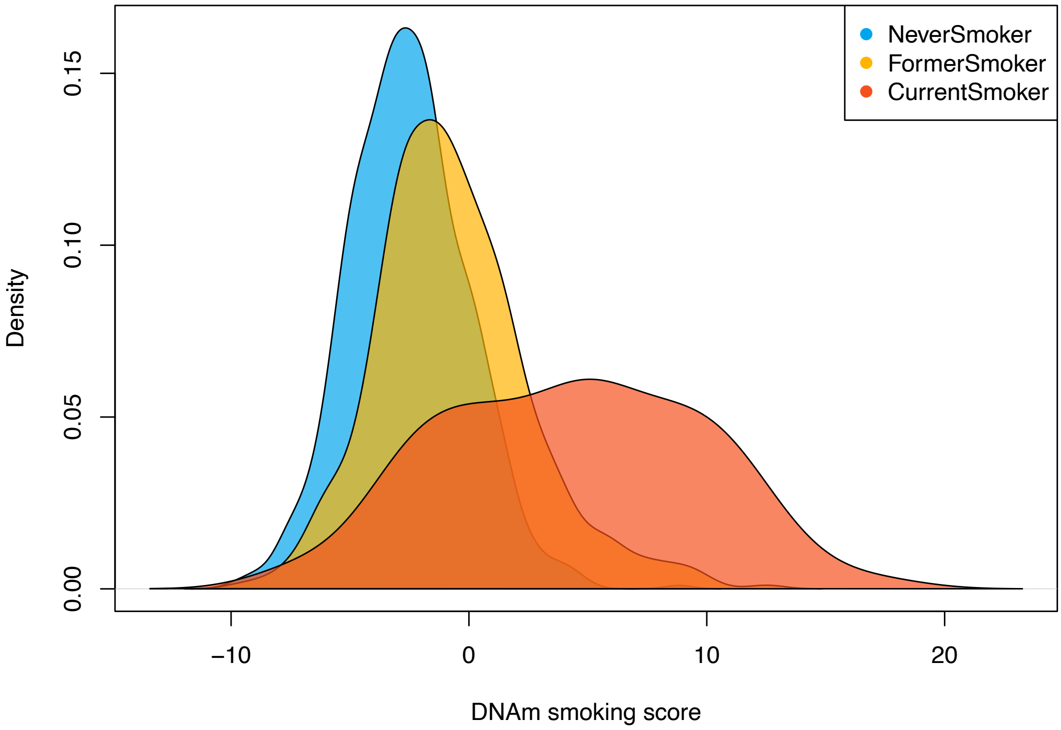

Supplementary Figure 15 Distribution of DNA methylation score.
DNA methylation score was built in current smokers versus never smokers of the Elliott et al study. DNA methylation score was then calculated for previous smokers. The overall distribution showed similarity comparing to the pSIN in the current smokers, previous smokers and never smokers in UKB.
