# Supplementary material for: Proteomic signatures of smoking and their associations with risk of incident diseases and mortality in diverse populations
Source: Nat Commun. 2025 Dec 24;17:928. doi: 10.1038/s41467-025-67656-x (PMC12830939; doi:10.1038/s41467-025-67656-x)
Supplement: Supplementary file 2 — Description of Additional Supplementary Files [file 41467_2025_67656_MOESM2_ESM.pdf]

Description of Additional Supplementary Files

File Name: Supplementary Data 1
Description: Baseline characteristics of UK Biobank participants stratified by smoking status (current vs never smokers). P-values were calculated by two-sided statistical tests.

File Name: Supplementary Data 2
Description: Baseline characteristics of China Kadoorie Biobank participants stratified by smoking status. P-values were calculated by two-sided statistical tests.

File Name: Supplementary Data 3
Description: List of 51 proteins selected by SHAP-based Boruta algorithm, with their annotations.

File Name: Supplementary Data 4
Description: Associations between self-reported smoking behaviour and proteomic smoking index (pSIN) in the UK Biobank stratified by smoking status. P-values were calculated by two-sided statistical tests.

File Name: Supplementary Data 5
Description: Associations between self-reported smoking behaviour and proteomic smoking index (pSIN) in the China Kadoorie Biobank stratified by smoking status. P-values were calculated by two-sided statistical tests.

File Name: Supplementary Data 6
Description: Genome-wide significant loci associated with pSIN identified in the UK Biobank.

File Name: Supplementary Data 7
Description: Genome-wide significant loci (lead significant SNP) associated with pSIN identified in the UK Biobank. P-values were calculated by two-sided statistical tests.

File Name: Supplementary Data 8
Description: Annotations of genes identified by GWAS and their functions.

File Name: Supplementary Data 9
Description: Overlap of pSIN-associated genes with previously reported GWAS in GWAS catalog. P-values were calculated by two-sided statistical tests.

File Name: Supplementary Data 10
Description: Genetic correlation estimates between pSIN and >1,400 traits using LD Score Regression. P-values were calculated by two-sided statistical tests. P-values were corrected for FDR multiple testing and was shown as p.adjust.

File Name: Supplementary Data 11
Description: Exposome-wide associations between pSIN and 176 environmental, lifestyle, and socioeconomic factors in the UK Biobank. P-values were calculated by two-sided statistical tests. P-values were corrected for FDR multiple testing and was shown as p.adjust.

File Name: Supplementary Data 12
Description: Associations between pSIN and blood biochemistry biomarkers, and clinical risk factor. P-values were calculated by two-sided statistical tests. P-values were corrected for FDR multiple testing and was shown as fdr_pval.

File Name: Supplementary Data 13
Description: Associations between pSIN and risk of health using multivariate Cox models in overall UK Biobank population. P-values were calculated by two-sided statistical tests.

File Name: Supplementary Data 14
Description: Validation of pSIN–disease and mortality associations in the China Kadoorie Biobank. P-values were calculated by two-sided statistical tests. P-values were corrected for FDR multiple testing and was shown as fdr_pval.

File Name: Supplementary Data 15
Description: Cumulative incidence rates at each 5-year age point for each disease by pSIN quartile (overall UK Biobank population).

File Name: Supplementary Data 16
Description: Numbers at risk for each disease at each 5-year age point by pSIN quartile (overall UK Biobank population).

File Name: Supplementary Data 17
Description: Associations between pSIN and risk of health using multivariate Cox models in current smokers of UK Biobank. P-values were calculated by two-sided statistical tests.

File Name: Supplementary Data 18
Description: Cumulative incidence rates at each 5-year age point for each disease by pSIN quartile (Current smokers in UK Biobank population).

File Name: Supplementary Data 19
Description: Numbers at risk for each disease at each 5-year age point by pSIN quartile (Current smokers in UK Biobank population).

File Name: Supplementary Data 20
Description: Associations between pSIN and risk of health using multivariate Cox models in previous smokers of UK Biobank. P-values were calculated by two-sided statistical tests.

File Name: Supplementary Data 21
Description: Cumulative incidence rates at each 5-year age point for each disease by pSIN quartile (previous smokers in UK Biobank population).

File Name: Supplementary Data 22
Description: Numbers at risk for each disease at each 5-year age point by pSIN quartile (previous smokers in UK Biobank population).

File Name: Supplementary Data 23
Description: Hazard ratios comparing low-risk versus high-risk previous smokers (defined by pSIN threshold) across health outcomes. P-values were calculated by two-sided statistical tests.

File Name: Supplementary Data 24
Description: Cumulative incidence comparisons for low-risk versus high-risk previous smokers at each 5-year age point across health outcomes.

File Name: Supplementary Data 25
Description: Numbers at risk for each disease at each 5-year age point for low-risk versus high-risk previous smokers across health outcomes.

File Name: Supplementary Data 26
Description: Hazard ratios comparing low-risk and high-risk current smokers (defined by pSIN threshold) across health outcomes. P-values were calculated by two-sided statistical tests.

File Name: Supplementary Data 27
Description: Cumulative incidence comparisons for low-risk and high-risk current smokers at each 5-year age point across health outcomes.

File Name: Supplementary Data 28
Description: Numbers at risk for each disease at each 5-year age point for low-risk versus high-risk current smokers across health outcomes.

File Name: Supplementary Data 29
Description: Literature cross-reference of the 51 proteins comprising pSIN against previous genome, epigenome, transcriptome, and single-protein studies of smoking.

File Name: Supplementary Data 30
Description: ICD-coded definitions used to identify prevalent and incident chronic diseases in the UK Biobank for the present analysis.
