# Supplementary material for: Proteomic signatures of smoking and their associations with risk of incident diseases and mortality in diverse populations
Source: Nat Commun. 2025 Dec 24;17:928. doi: 10.1038/s41467-025-67656-x (PMC12830939; doi:10.1038/s41467-025-67656-x)
Supplement: Supplementary file 4 — Reporting Summary [file 41467_2025_67656_MOESM4_ESM.pdf]

nature portfolio

Corresponding author(s): Sihao Xiao, Cornelia van Duijn

Last updated by author(s): Oct 27, 2025

Reporting Summary

Nature Portfolio wishes to improve the reproducibility of the work that we publish. This form provides structure for consistency and transparency in reporting. For further information on Nature Portfolio policies, see our Editorial Policies and the Editorial Policy Checklist.

Statistics

For all statistical analyses, confirm that the following items are present in the figure legend, table legend, main text, or Methods section.

n/a	Confirmed	
☐	☒	The exact sample size (n) for each experimental group/condition, given as a discrete number and unit of measurement
☐	☒	A statement on whether measurements were taken from distinct samples or whether the same sample was measured repeatedly
☐	☒	The statistical test(s) used AND whether they are one- or two-sided<br>*Only common tests should be described solely by name; describe more complex techniques in the Methods section.*
☐	☒	A description of all covariates tested
☐	☒	A description of any assumptions or corrections, such as tests of normality and adjustment for multiple comparisons
☐	☒	A full description of the statistical parameters including central tendency (e.g. means) or other basic estimates (e.g. regression coefficient) AND variation (e.g. standard deviation) or associated estimates of uncertainty (e.g. confidence intervals)
☐	☒	For null hypothesis testing, the test statistic (e.g. F, t, r) with confidence intervals, effect sizes, degrees of freedom and P value noted<br>*Give P values as exact values whenever suitable.*
☒	☐	For Bayesian analysis, information on the choice of priors and Markov chain Monte Carlo settings
☒	☐	For hierarchical and complex designs, identification of the appropriate level for tests and full reporting of outcomes
☐	☒	Estimates of effect sizes (e.g. Cohen's d, Pearson's r), indicating how they were calculated

Our web collection on statistics for biologists contains articles on many of the points above.

Software and code

Policy information about availability of computer code

Data collection	No software used. All data was already collected.
Data analysis	All statistical analyses were carried out using Python v.3.9.16 and R v.4.2.2. Analysis code are available at https://github.com/xiaosihao/ProteomicSmoking

For manuscripts utilizing custom algorithms or software that are central to the research but not yet described in published literature, software must be made available to editors and reviewers. We strongly encourage code deposition in a community repository (e.g. GitHub). See the Nature Portfolio guidelines for submitting code & software for further information.

Data

Policy information about availability of data

All manuscripts must include a data availability statement. This statement should provide the following information, where applicable:

- Accession codes, unique identifiers, or web links for publicly available datasets
- A description of any restrictions on data availability
- For clinical datasets or third party data, please ensure that the statement adheres to our policy

UK Biobank data are available through a procedure described at: https://www.ukbiobank.ac.uk/enable-your-research.
The China Kadoorie Biobank (CKB) is a global resource for the investigation of lifestyle, environmental, blood biochemical and genetic factors as determinants of common diseases. The CKB study group is committed to making the cohort data available to the scientific community in China, the UK, and worldwide to advance

knowledge about the causes, prevention and treatment of disease. For detailed information on what data is currently available to open access users and how to apply for it, please visit: https://www.ckbiobank.org/data-access. A research proposal will be requested to ensure that any analysis is performed by bona fide researchers. Researchers who are interested in obtaining additional information or data that underlines this paper should contact ckbaccess@ndph.ox.ac.uk. For any data that is not currently available to open access, researchers may need to develop formal collaboration with the CKB study group.

Research involving human participants, their data, or biological material

Policy information about studies with human participants or human data. See also policy information about sex, gender (identity/presentation), and sexual orientation and race, ethnicity and racism.

Reporting on sex and gender	After regressing out recruitment age and sex, the proteomic data were further processed by first rescaling to the value between 0 and 1 and then centring on the median. To address the concern regarding the potential residual signal of age and sex in the proteomic expression data after linear regression, we performed additional analyses to rigorously evaluate the effectiveness of the regression process. Specifically, we trained gradient boosting models to assess whether any predictive signal for age or sex remained in the regressed data. For age, we trained regression models to predict age using each protein in the regression dataset. The results showed a mean R^2 of -0.0042 (SD = 0.0089), indicating that the models performed no better than random chance. This suggests that the linear regression successfully removed age-related signals from the proteomic data. For sex, we trained classification models to predict sex using each protein in the regressed dataset. The models achieved a mean AUC of 0.51 (SD = 0.017), which is equivalent to random guessing. This confirms that sex-related signals were effectively removed by the regression process.<br>We also compared the performance of the model in males and females in the UKB test dataset. The result showed slightly higher performance in females compared to males, with an AUC of 0.96 (SD=1.11*10-16) and 0.94 (SD=2.06*10-296), respectively.<br>We further compared the performance of the trained model between males and females in CKB. The model showed a slightly higher AUC for males (AUC=0.90, SD=2.06*10-296; F1=0.98, SD=4.44*10-16; AP=0.91, SD=2.06*10-296; AP=0.77, SD=2.22*10-16) comparing to females (AUC=0.89, SD=2.06*10-296; F1=0.98, SD=2.22*10-16; AP=0.90, SD=1.11*10-16; AP=0.74, SD=2.22*10-16).
Reporting on race, ethnicity, or other socially relevant groupings	For associations between smoking history and pSIN, models were adjusted for basic socioeconomic factors, including recruitment centre, ethnicity, education years, and the Townsend deprivation index.<br>For exposome-wide association analysis (all available social-economic and lifestyle variables available in UKB), models were only adjusted for the most basic recruitment centre, ethnicity and smoking status to allow exploration of a wide range of potential associations between the exposome and pSIN without masking potential signals by controlling for too many variables.<br>Social-economic and lifestyle confounding factors were chosen based on previous literatures54–56.
Population characteristics	we used a subset of the UK Biobank (UKB) with available measurements of the plasma proteome. The study population consisted of 43,914 participants, with 4,732 self-reported as current regular cigarette smokers, and 23,778 as never smokers.We further validated the accuracy of our proteomic-based smoking score in participants from the China Kadoorie Biobank (n=3,977).
Recruitment	The UKB is a prospective cohort study which includes 502,505 participants recruited between 2006 and 2010. Further information on UKB recruitment and data collection has been published previously (www.ukbiobank.ac.uk/media/gnkeyh2q/study-rationale.pdf)<br>The China Kadoorie Biobank (CKB) is a prospective cohort study which includes 512,724 participants recruited from 10 geographically diverse regions between 2004 to 2008. Information on CKB recruitment and data collection has been previously reported (doi.org/10.1093/ije/dyr120)
Ethics oversight	UK Biobank data use (Project Application Number 61054) was approved by the UK Biobank according to their established access procedures. UK Biobank has approval from the North West Multi-centre Research Ethics Committee (MREC) as a Research Tissue Bank (RTB), and as such researchers using UK Biobank data do not require separate ethical clearance and can operate under the RTB approval. The China Kadoorie Biobank (CKB) complies with all the required ethical standards for medical research on human subjects. Ethical approvals were granted and have been maintained by the relevant institutional ethical research committees in the UK and China.

Note that full information on the approval of the study protocol must also be provided in the manuscript.

Field-specific reporting

Please select the one below that is the best fit for your research. If you are not sure, read the appropriate sections before making your selection.

☐ Life sciences ☒ Behavioural & social sciences ☐ Ecological, evolutionary & environmental sciences

For a reference copy of the document with all sections, see nature.com/documents/nr-reporting-summary-flat.pdf

Behavioural & social sciences study design

All studies must disclose on these points even when the disclosure is negative.

Study description	we developed a proteomic-based smoking profile in the UK Biobank (n=43,914) using machine learning. We further validated the accuracy of our proteomic-based smoking score in participants from the China Kadoorie Biobank (n=3,977). We then identified the genetic and exposome factors influencing the smoking proteomic score. Finally, we examined the associations between our proteomic-based smoking score and clinical risk factors, blood-based biomarkers, risks of 27 major diseases and mortality in the UK Biobank.
Research sample	Our study uses secondary data from 43,914 UK Biobank (UKB, 54% female, age range: 39-71 years), 3,977 Chinese (CKB) participants in an ischemic heart disease (IHD) case-cohort study (54% female, age range: 30-78 years). We chose the UKB, and CKB, or the breadth of exposures, phenotypes, incident disease diagnosis data, and biological data available in each, allowing for comprehensive and integrative modeling. We used previously collected self-report questionnaire data, data from clinical interviews, biochemical measures from blood samples collected at baseline, and hospital diagnosis and mortality information from linked inpatient and mortality register data. UKB proteomics samples were randomly selected to be roughly representative of the full UKB cohort. CKB proteomics samples are not representative of the full CKB cohort due to the nested case-cohort design.
Sampling strategy	The final sample included 43,914 UKB participants, and 3,977 CKB participants. We randomly split the UKB cohort into 70% training and 30% test sets to develop the proteomic smoking index. In the training phase, we trained the model to predict smoking status using the normalized expression of 2,897 proteins from the Olink Explore 3027 panel.<br>Models were then validated in the CKB participants. Associations between proteomic smoking index and health outcomes and clinical phenotypes were tested in the full UKB and CKB participants. Subsampling within each cohort to select participants for proteomic analysis was performed previously by the cohorts and not by our study team. UKB proteomics samples were randomly selected to be roughly representative of the full UKB cohort. CKB proteomics samples were selected using a nested case-cohort design to be powered for ischemic heart disease analyses. Power analyses were not conducted for this study - we used all participants with proteomics data available in each cohort. With the exception of UKB, where we excluded roughly 7,000 participants who were not randomly selected and instead selected by pharma partners to be enriched for different disease phenotypes.
Data collection	Participants were recruited to the UKB between 2006-2010. Further information on UKB recruitment and data collection has been published previously (https://www.ukbiobank.ac.uk/media/gnkeyh2q/study-rationale.pdf). CKB participants were recruited from ten geographically diverse (five rural and five urban) areas across China during 2004-2008. Information on CKB recruitment and data collection has been previously reported (https://doi.org/10.1093/ije/dyr120). No new data were collected from UKB, or CKB participants for this study. Researchers in our study were not blinded to the study hypothesis.
Timing	Baseline UKB collection took place from March 15 2006 until September 27 2010. Follow up mortality and incident disease data were collected until December 2 2022, leaving 11-15 years of follow up in the UKB. Baseline CKB collection took place from 2004-2008 (precise dates not reported in dataset).
Data exclusions	We restricted our UKB participants to those with Olink Explore data available at baseline who were randomly sampled from the main UKB population. We restricted our CKB sample to those participants with Olink Explore data available.
Non-participation	No Participants dropped out during the course of our study.
Randomization	Participants were randomly assigned to training and test sets for completing machine learning analyses

Reporting for specific materials, systems and methods

We require information from authors about some types of materials, experimental systems and methods used in many studies. Here, indicate whether each material, system or method listed is relevant to your study. If you are not sure if a list item applies to your research, read the appropriate section before selecting a response.

Materials & experimental systems

n/a	Involved in the study
☒	☐ Antibodies
☒	☐ Eukaryotic cell lines
☒	☐ Palaeontology and archaeology
☒	☐ Animals and other organisms
☒	☐ Clinical data
☒	☐ Dual use research of concern
☒	☐ Plants

Methods

n/a	Involved in the study
☒	☐ ChIP-seq
☒	☐ Flow cytometry
☒	☐ MRI-based neuroimaging

Plants

Seed stocks

Report on the source of all seed stocks or other plant material used. If applicable, state the seed stock centre and catalogue number. If plant specimens were collected from the field, describe the collection location, date and sampling procedures.

Novel plant genotypes

Describe the methods by which all novel plant genotypes were produced. This includes those generated by transgenic approaches, gene editing, chemical/radiation-based mutagenesis and hybridization. For transgenic lines, describe the transformation method, the number of independent lines analyzed and the generation upon which experiments were performed. For gene-edited lines, describe the editor used, the endogenous sequence targeted for editing, the targeting guide RNA sequence (if applicable) and how the editor was applied.

Authentication

Describe any authentication procedures for each seed stock used or novel genotype generated. Describe any experiments used to assess the effect of a mutation and, where applicable, how potential secondary effects (e.g. second site T-DNA insertions, mosiacism, off-target gene editing) were examined.
